# Toward the understanding of DSG2 and CD46 interaction with HAdV-11 fiber, a super-complex analysis

**DOI:** 10.1128/jvi.00910-23

**Published:** 2023-11-03

**Authors:** Gregory Effantin, Marc-André Hograindleur, Daphna Fenel, Pascal Fender, Emilie Vassal-Stermann

**Affiliations:** 1 Université Grenoble Alpes, CNRS, CEA, IBS, Grenoble, France; International Centre for Genetic Engineering and Biotechnology, Trieste, Italy

**Keywords:** adenoviruses, receptors, virus-host interactions, electron microscopy

## Abstract

Among human species B adenovirus, HAdV-11, has the particular feature of exploiting two primary receptors, desmoglein 2 (DSG2) and CD46, to mediate cell attachment. This serotype represents a particularly promising class of oncolytic viral vaccines. For instance, Enadenotucirev, a species B HAdV-11/HAdV-3 chimeric adenoviral vector, demonstrated preclinical tumor-selective cytotoxicity. Understanding the mechanism of HAdV-11 entry is a major challenge for the development of powerful vectors. Interactions between HAdV-11 and CD46 are well-characterized and clearly depend on the fiber protein, but the mechanism whereby HAdV-11 engages DSG2 has been still unresolved. In this study, we described a new cryo-EM structure of the HAdV-11 fiber in complex with DSG2. Kinetic interaction studies demonstrated that the binding stability of the HAdV-11 in complex with DSG2 was significantly lower than with CD46. Furthermore, competition assays with pseudotyped viruses suggested that the interaction of the HAdV-11 fiber with DSG2 was not required for infection but that DSG2 could serve as a receptor in the absence of CD46. Unexpectedly, we succeeded to isolate a HAdV-11 fiber/DSG2/CD46 “super complex” showing that these two receptors could simultaneously bind to the same trimeric HAdV-11K. Our work deciphers the use of two independent receptors by HAdV-11 from a biochemical and structural point of view, which can have an impact for future vectors design.

**IMPORTANCE:**

The main limitation of oncolytic vectors is neutralization by blood components, which prevents intratumoral administration to patients. Enadenotucirev, a chimeric HAdV-11p/HAdV-3 adenovirus identified by bio-selection, is a low seroprevalence vector active against a broad range of human carcinoma cell lines. At this stage, there’s still some uncertainty about tropism and primary receptor utilization by HAdV-11. However, this information is very important, as it has a direct influence on the effectiveness of HAdV-11-based vectors. The aim of this work is to determine which of the two receptors, DSG2 and CD46, is involved in the attachment of the virus to the host, and what role they play in the early stages of infection.

## INTRODUCTION

Human adenoviruses (HAdVs) are common pathogens associated with a range of clinical illnesses, including conjunctivitis, upper and lower respiratory tract diseases, gastroenteritis, hemorrhagic cystitis, hepatitis, and myocarditis (1). Currently, more than 110 HAdV types have been identified and grouped into seven species, A through G (http://hadvwg.gmu.edu/), based on genetic and biological characteristics. Among species B, HAdV-11 stands out for its ability to use similarly two different protein receptors desmoglein 2 (DSG2) and CD46 to mediate cell binding (2, 3). This serotype was first isolated from a stool of a child diagnosed with poliomyelitis in 1954 (4, 5). It has been reported to be associated with upper and lower respiratory illnesses; hemorrhagic cystitis in children and young adults, especially in renal transplant recipients and occasionally with endemic hemorrhagic conjunctivitis. However, HAdV-11 infections have not been reported for over 20 years explaining the low prevalence of neutralizing antibodies to HAdV-11 in the general population compared to other adenovirus serotypes (6, 7).

Species B serotypes represent a particularly promising class of oncolytic viral vaccines. Indeed, the presence of relevant receptors on host cells is crucial for the efficiency of viral vectors. Remarkably, the distinctive tropism of HAdV-11 is an advantage for clinical applications of vectors derived from this serotype as it allows targeting of colon cancer cells and to a lesser extent renal and ovarian cells (8). CD46 is localized to both apical and basolateral membranes of polarized epithelial cells (9, 10). It is reported to be upregulated in some human tumors (11) and distributed throughout the surface of cancer cells (12), making it more accessible. In the same way, some epithelial cancers are known to highly upregulate the expression of DSG2 (13, 14). Unlike CD46, DSG2 is a predominantly basolateral cell adhesion component. Based on these beneficial characteristics, EnAdenotucirev (EnAd), a chimera of adenovirus type 3 (HAdV-3) and type 11p (HAdV-11p), was selected upon directed evolution for its potent tumor-selective cytotoxicity. This chimera consists of HAdV-11p, with a nearly complete E3 region deletion, a smaller deletion in the E4 region, and a chimeric HAdV-3/HAdV-11p E2B region (15). This oncolytic adenovirus is currently under clinical evaluation, one in combination with chemoradiation for the treatment of advanced rectal cancer (NCT03916510) and another combined with ICB for advanced solid tumors (NCT02636036) (8, 16). Despite its high specificity for carcinoma cell lines, the accurate mechanism of EnAd’s selectivity is still under investigation.

Understanding of how viral entry proteins interact with their host-cell receptors offers important opportunities for the development of novel therapeutics and vaccines. Interactions between HAdV-11 and CD46 are well-characterized and clearly depend on the fiber protein (17, 18) but contrary to HAdV-3 and HAdV-7 little is known about the mechanism whereby HAdV-11 engages DSG2 (19 – 21).

In this study, we elucidate the molecular basis for HAdV-11 recognition of DSG2 by resolving the structure of extracellular domains of DSG2 in complex with the HAdV-11 fiber knob protein. The structure shows that DSG2 and CD46 engage different HAdV-11 residues and interfaces without overlapping. Interestingly, we also demonstrate, for the first time, that under specific conditions, HAdV-11 is able to simultaneously bind the two receptors CD46 and DSG2 at once.

## RESULTS

### Kinetic analysis of HAd11 knob binding to CD46 and DSG2

**Fig 1:**
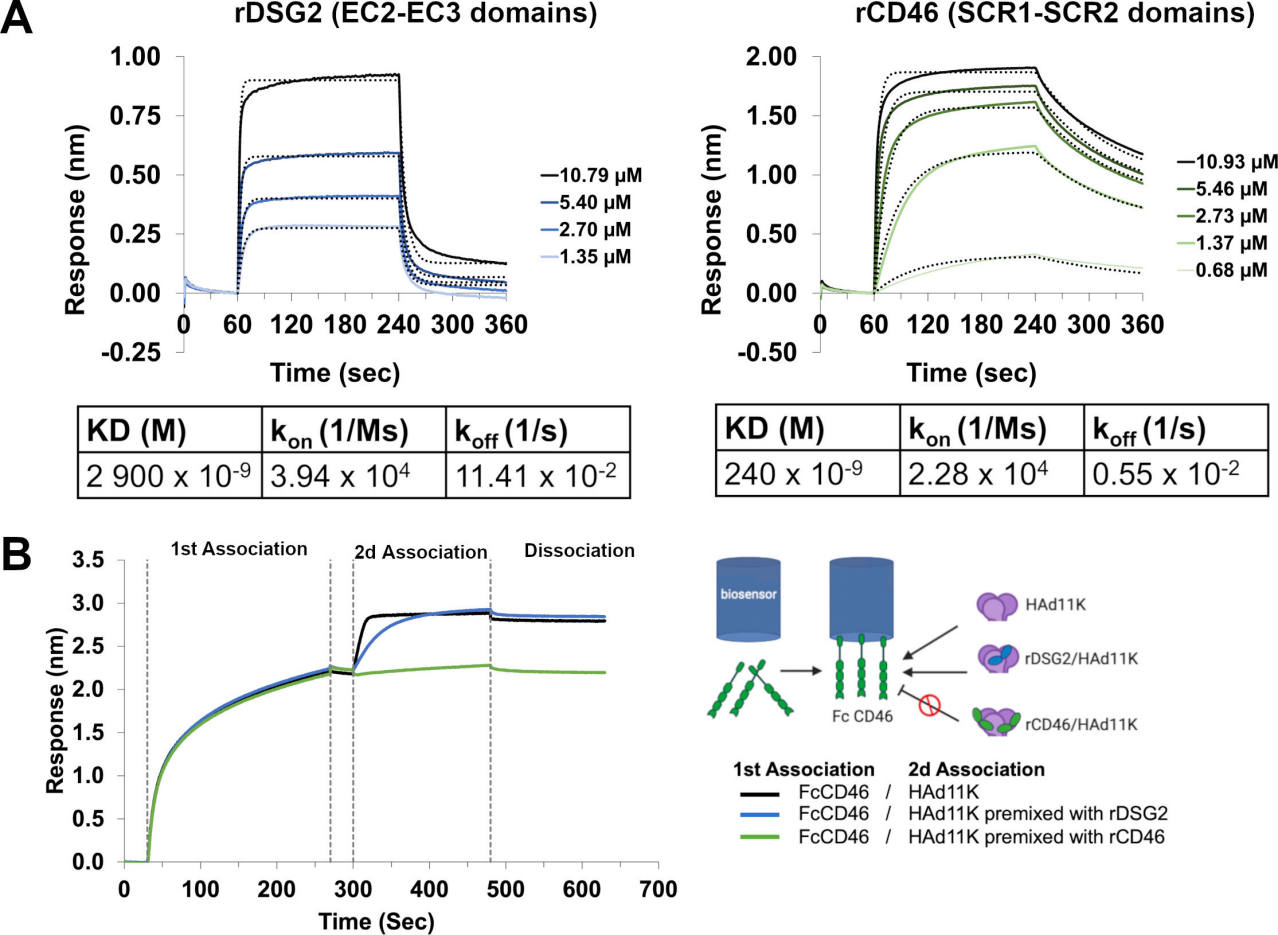
(A) Biolayer interferometry analyses of HAd11K binding to its receptors. Recombinant HAd11K, 100 µg/mL in 10 mM sodium acetate, pH 4, was coupled onto an Octet Amine Reactive Second-Generation (AR2G) biosensor. For kinetics analysis, rDSG2 (EC2-EC3 domains) or rCD46 (SCR1-SCR2 domains) were diluted in the running buffer (HBS-P) (Cytiva) with 3 mM CaCl_2_. The concentrations tested were 0.7, 1.4, 2.7, 5.4, and 10.8 µM. All the experiments were performed at room temperature, including association for 180 s, and dissociation for 120 s. The dotted lines represent fits of the raw data (solid lines). They were used to obtain KD, kon, and koff values. (**B**) Competitive behavior between DSG2 and CD46 was determined using a premix assay. FcCD46 was first captured using Octet Protein A Biosensors (1st Association). HAd11K pre-complexed or not with rDSG2 or rCD46 was allowed to bind to the immobilized FcCD46 (2d Association). Black line, HAd11K; Blue line, HAd11K premixed with rDSG2; green line, HAd11K premixed with rCD46.

DSG2 and CD46 are both able to bind to HAdV-11. Presumably, these two receptors may be competitive or independent, or they may cooperate with each other to bind the virus. Biolayer interferometry (BLI) was used to study the affinity and kinetics of the interaction between the human HAdV-11 fiber knob (HAd11K) and recombinant DSG2 or CD46 extracellular domains. Wild-type fiber knobs of HAd11K were expressed in fusion with histidine-tag in bacteria, and the trimeric forms were purified by nickel affinity chromatography and size exclusion. rDSG2 consists of the EC2–EC3 extracellular domain of DSG2, identified as the minimum essential region for interaction with HAd3K and HAd7K. rCD46 contains the knob-interacting CD46 domains SCR1 and SCR2. HAd11K was covalently coupled to the biosensor surface, and various concentrations of rCD46 or rDSG2 were injected. This configuration measures the interaction of one molecule of receptor to a single-binding site on the knob without being influenced by potential avidity effects. The values for the kinetic-binding constants were determined by globally fitting the data. The association rate constant (*k*_on_) determined for rCD46 was 2.28 × 10^4^ M^−1^ s^−1^, and the dissociation constant (*k*_off_) was found to be 0.005 s^−1^, resulting in an equilibrium dissociation constant (*K*_D_) of 243 nM. The measured *k*_on_ for rDSG2 binding was 3.94 × 10^4^ M^−1^ s^−1^ similar to that of rCD46. However, the *k*_off_ obtained for the rDSG2 was more than 20-fold higher than for the rCD46, with a *k*_off_ of 0.114 s^−1^ (Fig. 1A). This provides evidence that the binding stability of the HAd11K-rDSG2 complex is significantly weaker than the HAd11K-rCD46 interactions. As a result, the *K*_D_ as determined from the ratio of these constants was 2,900 nM, a more than 10-fold lower affinity than that of rCD46 with the Ad11 fiber knob.

BLI was used to conduct competitive binding experiments between soluble rCD46 or rDSG2 and Fc-CD46, containing the four SCR domains fused with Fc-fragment. Soluble rCD46 compete with HAd11K binding to immobilized Fc-CD46. A 14-fold excess of soluble rCD46 abolished almost all of the initial rCD46/HAd11K binding interaction (Fig. 1B). Conversely, under the same conditions, soluble rDSG2 cannot disrupt the native HAd11K/FcCD46 interaction. Altogether, these data confirm that HAd11K binds with a good affinity to CD46 and that the weaker DSG2 interaction poorly interfere with this binding mechanism. This suggests an independent way of binding HAd11K to these two receptors.

### Interaction of HAd11 knob with DSG2 is not required for HAdV-11 infection

#### HAdV-5/11 entry is highly competed by rCD46 and poorly by rDSG2

**Fig 2:**
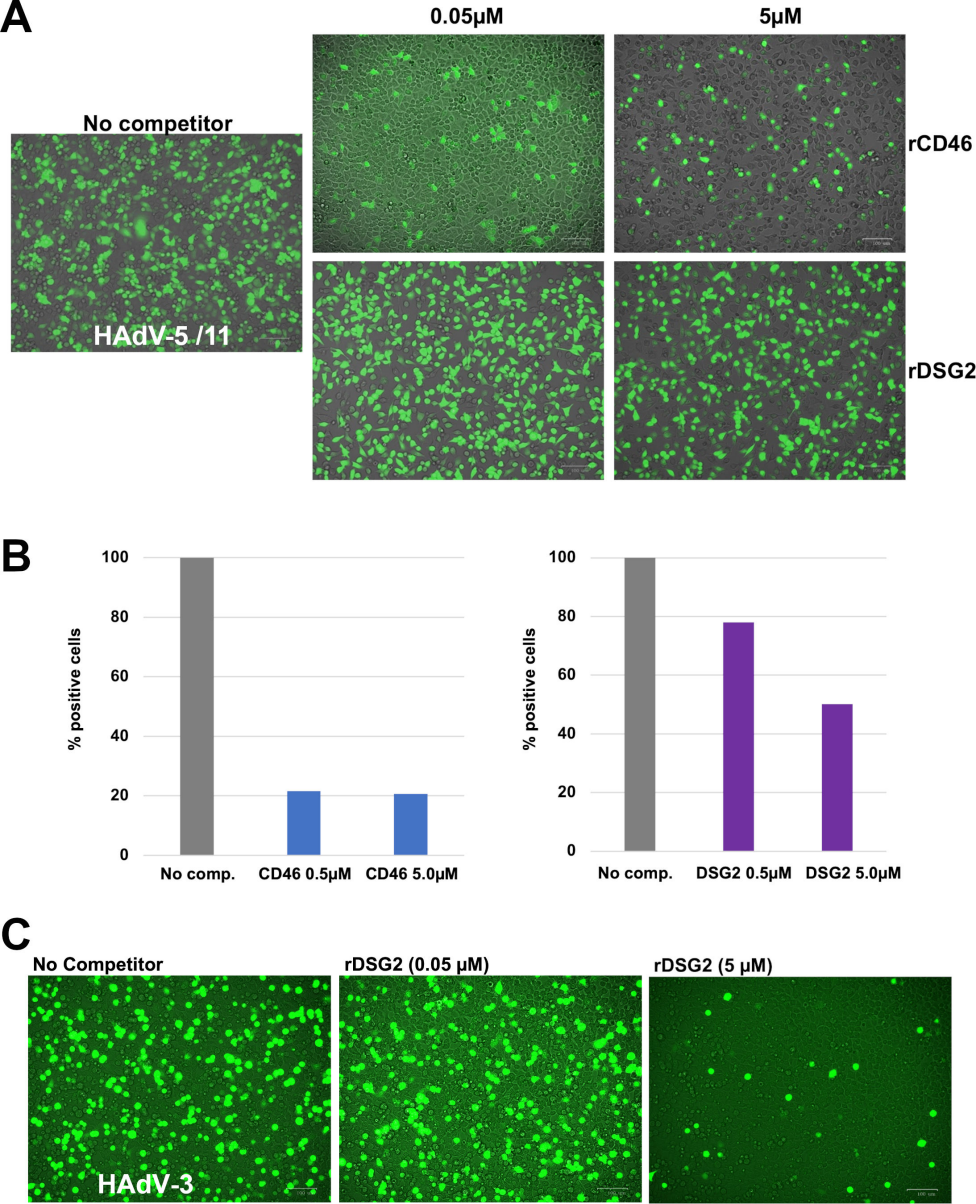
HAdV-5/11 or HAdV-3 infection competition studies with either soluble rDSG2 or rCD46 receptor. (**A**) Visualization of HAdV-5/11 encoding GFP infected cell 24H post infection using ZOE. (**B**) Quantification of HAdV-5/11 encoding GFP expressing cells by FACS. The percentage of infected cells is calculated using the cell infected by HAd5/11V w:o competitor as 100% of infection. (**C**) Control experiment with HAdV-3 encoding GFP and rDSG2 as competitor.

In order to investigate the respective contribution of CD46 and DSG2 in virus entry, competition assays were performed with HAdV-5 pseudotyped with HAdV-11 fibers (HAdV-5/11). HAdV-5/11 encoding GFP was incubated with either soluble rCD46 (SCR1 SCR2 domains) or rDSG2 (EC2 EC3 domains) prior to addition to HeLa cells. GFP expression was imaged 24H latter by immuno-fluorescence. If a clear reduction of GFP-positive cells was observed when rCD46 was used at 0.5 and 5 µM, this effect seemed to be modest with rDSG2 at the same concentrations (Fig. 2A). The functionality of rDSG2 cannot be incriminated since in a control experiment, a strong inhibition of HAdV-3 encoding GFP was observed even at lower concentrations (Fig. 2C). Moreover, this control experiment confirms that HAdV-3 uses DSG2 as main attachment receptor.

FACS analysis was then performed on the same cells detached from the support by trypsin to assess the inhibitory potential of each competitor using cells infected by HAdV-5/11 without competitor as the 100% of infection. The trend observed in the immunofluorescence study was confirmed with a reduction of 80% of GFP-positive cells with rCD46 at 0.5 µM while it was only of 22% with rDSG2 at the same concentration (Fig. 2B). A higher concentration of competitor (5 µM) did not enable a better competition with rCD46, and a plateau was reached. If the addition of rDSG2 at 5 µM allowed for a better inhibitory effect at 50% instead of 22% at 0.5 µM, it is still far less than with rCD46 at a lower concentration (Fig. 2B, right panel). Altogether, these results suggest that CD46 is the main cellular receptor for HAdV-5/11 entry, but DSG2 could, nevertheless, play an accessory role. These results are in line with the aforementioned biochemical observations.

#### DSG2 serves as a receptor in the absence of CD46

Both biochemical and cellular data suggest that CD46 is the main entry receptor for HAdV-5/11. The role played by DSG2 in viral entry remains, therefore, uncertain. To address this point, a CHO-K1 cell line stably expressing the full-length human DSG2 (CHO-DSG2) was generated. A FACS analysis experiment was then set up to see whether the expression of this receptor can restore HAdV-5/11 binding and entry as compared to CHO-K1^wt^ cells lacking both DSG2 and CD46.

**Fig 3:**
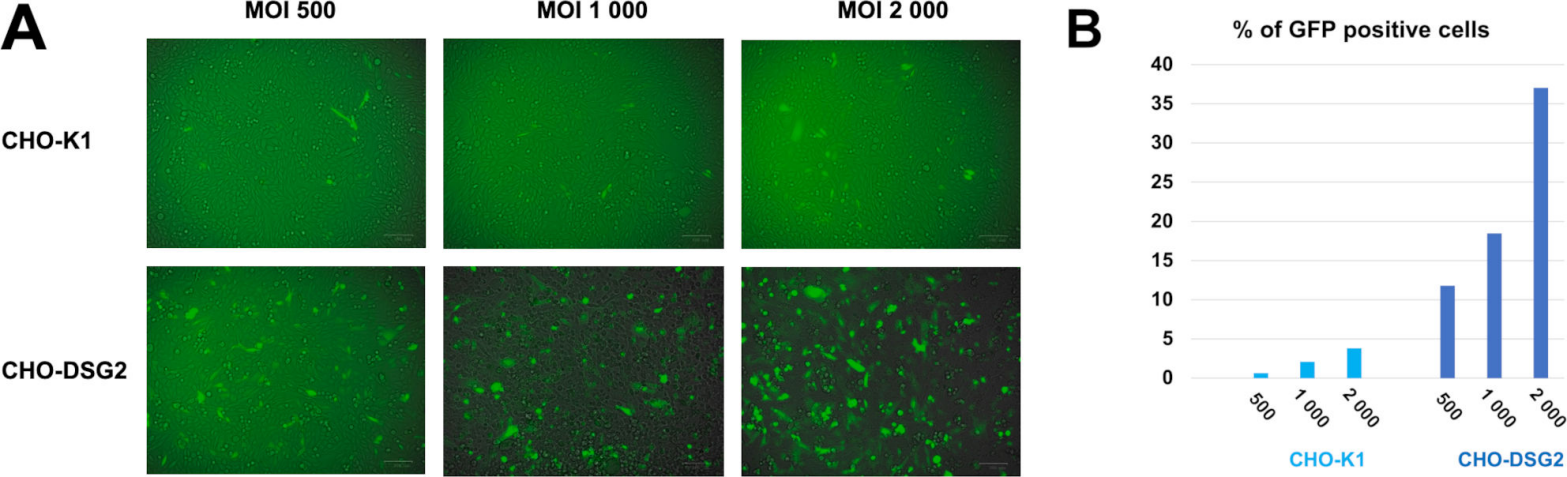
HAdV-5/11 entry in CHO-K1 and CHO-DSG2. (**A**) HAdV-5/11 was incubated at the indicated MOI with either CHO-K1 or CHO-DSG2. One day post infection, GFP expression was observed using ZOE. (**B**) Quantification of GFP-expressing cells was performed using FACS using non-infected cells to determine the threshold of GFP-positivity.

We observed that HAdV-5/11 can bind and enter CHO cells expressing human DSG2 but did not significantly enter CHO-K1 cells lacking this receptor (Fig. 3). It can be concluded that DSG2 expression alone can mediate HAdV-5/11 entry and, thus, overcome the limitation due to the lack of CD46 expression. This result highlights that even if CD46 seems to be the main receptor for HAdV-11 entry, its expression is not strictly required for viral infection when DSG2 is expressed.

### HAd11K/rDSG2 complex formation in solution and purification

**Fig 4:**
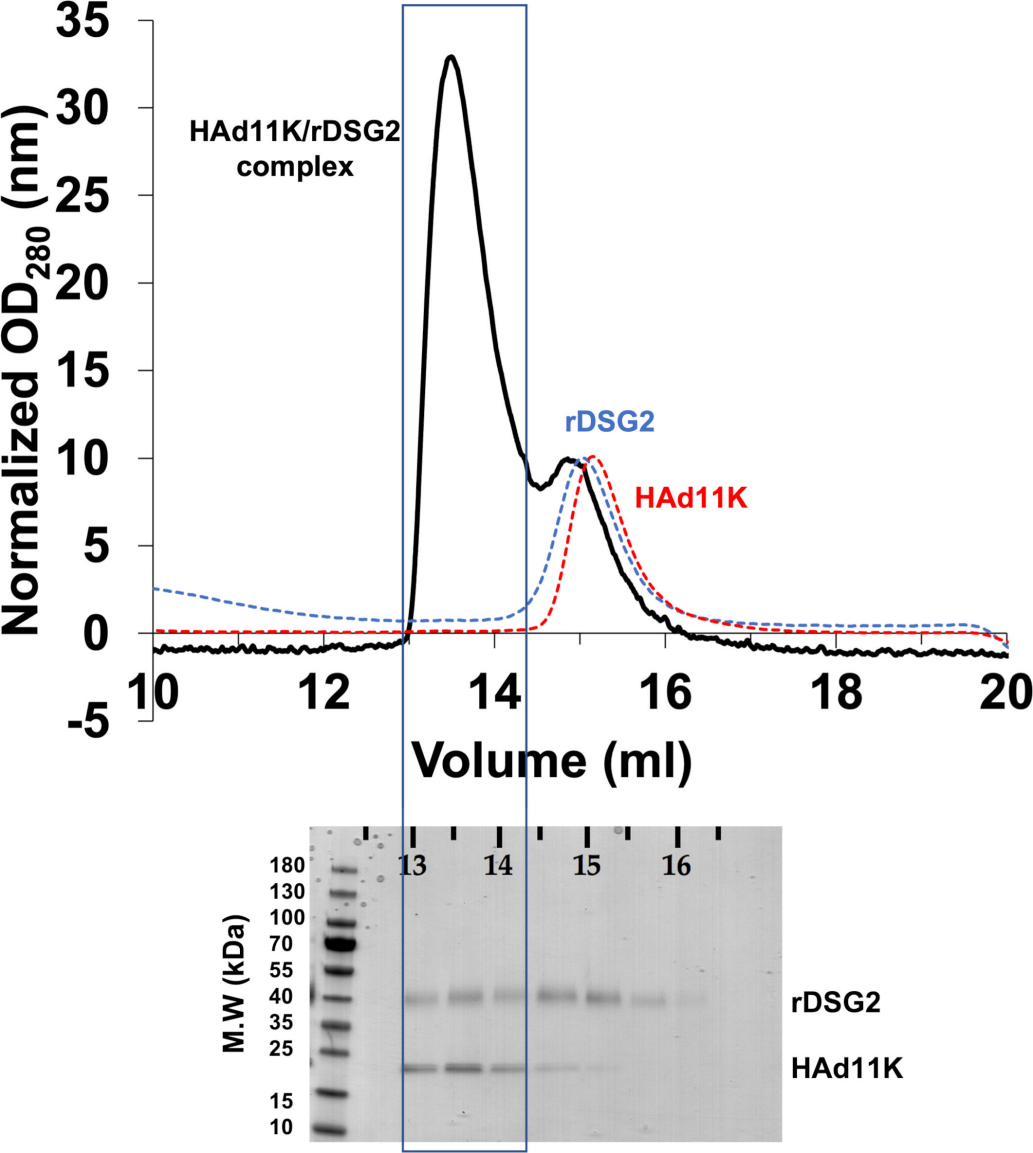
Analytical size exclusion chromatograms of HAd11K in the presence (dark line) and absence (dashed red line) of rDSG2. HAd11K was preincubated overnight with or without an equimolar concentration of DSG2, prior injection onto a Superdex S200 column. A chromatogram of rDSG2 alone is shown as a dashed blue line. Fractions corresponding to the black chromatogram were separated by SDS-PAGE, followed by Coomassie blue staining.

The capacity of purified rDSG2 and HAd11K to form a complex in solution was then investigated. To do this, rDSG2 and HAd11K were co-incubated in a calcium-containing buffer overnight at 4°C prior to loading on a gel filtration column (Superdex 200). Eluted fractions were analyzed on an SDS-PAGE gel. When rDSG2 or HAd11K were run individually on the Superdex column, the elution profile shows peaks eluted around 15 mL (Fig. 4). When equimolar amounts of rDSG2 and HAd11K were mixed, the elution profile shows that a peak is eluted at 13.5 mL suggesting the presence of a HAd11K/ rDSG2 complex.

### Structure determination of the HAd11 knob in complex with rDSG2

**Fig 5:**
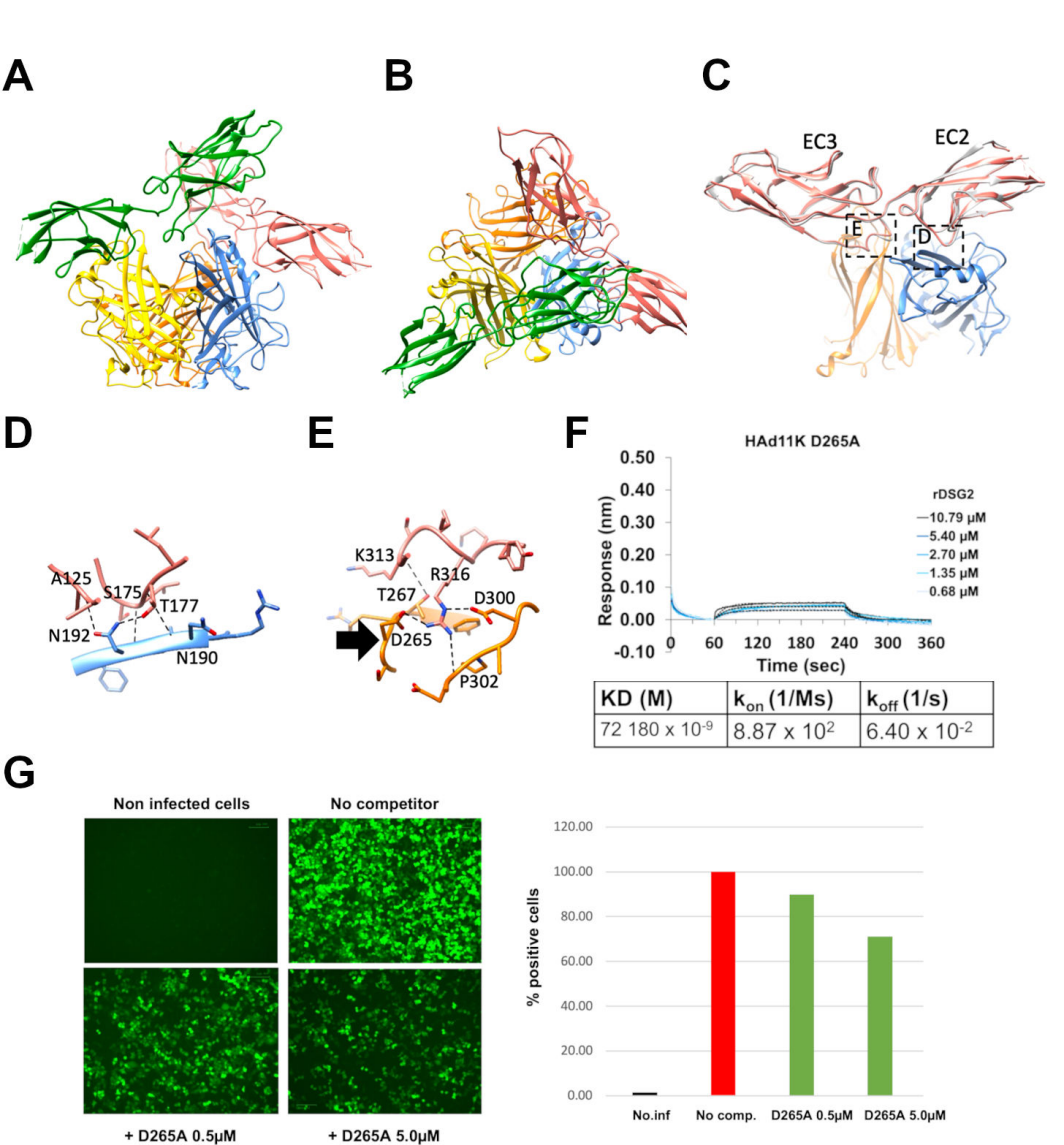
Structure of HAd11K/ rDSG2 complex and identification of critical binding regions. (A and B) Ribbon representation of the HAd11K trimer (orange, yellow, and blue subunits) in complex with two rDSG2 molecules (salmon and green subunits) in side (**A**) and top views (**B**). (C) Overview of the two interaction sites of rDSG2 on two different HAd11K monomers using the same color code as in A and B. Superimposed onto the rDSG2 molecule (salmon) is a ribbon representation of a rDSG2 molecule (gray), which was determined in complex with the HAd7 knob. (D and E) Zoomed views on the two contact zones between rDSG2 and HAd11K highlighted in (**C**). Putative hydrogen bonds and salt bridges are shown in dotted black lines. (**F**) Biolayer interferometry sensorgram showing mutated HAd11K (D265A) binding to different concentrations of DSG2 and KD determination. (**G**) Competition on HeLa cells of HAdV-5/11 preincubated with mutated HAd11K D265A. Visualization of GFP-infected cell 24H post infection using ZOE (left panel) and quantification of GFP-expressing cells by FACS (right panel). The percentage of infected cells is calculated using the cell infected by HAd5/11V w:o competitor as 100% of infection.

Based on the successful strategy of our previous work (19, 21), the structure of HAd11K/ rDSG2 was solved by cryoEM. During the image analysis, two main populations of particles consisting of the HAd11K in complex with either one or two rDSG2 modules were identified and two 3D reconstructions were obtained at 3.5 and 3.2 Å resolution for HAd11K in complex with one or two rDSG2, respectively (Fig. S1). The binding of rDSG2 to HAd11K is very similar in both populations meaning that the binding of a second rDSG2 is possible without interfering with the first rDSG2 (Fig. 5A and B). Moreover, in the population with two rDSG2 attached to the HAd11K, each rDSG2 binds HAd11K in the same way with the same interfacing residues (Table S2). rDSG2 consists of two modules, EC2 and EC3, and each one interacts with a different monomer of HAd11K (Fig. 5C). The tips of two loops in EC2 (around residue A125 and residues S175, T177, respectively) are in close contact with residues located on the apex of the HAd11K (in particular residues N190 and N192). Several putative hydrogen bonds are predicted in this region which likely contributes to the binding (Fig. 5D and Fig. S1). Regarding the interaction of the EC3 module of rDSG2 with HAd11K, the loop region centered around R316 of EC3 fits snugly in an apical region of HAd11K defined by two loops centered around D265 and D300, respectively. Several hydrogen bonds and salt bridges are also predicted in this area (Fig. 5E and Fig. S1). The interacting regions of rDSG2 on HAd11K are strikingly similar to the one found for HAd7K (19) (Fig. 5C) and HAd3K (21), two other adenoviruses which are using DSG2 as their cellular receptor. Indeed, HAd11K and HAd7K are very similar in sequence (92.9% identity), and the interacting residues with rDSG2 are very well conserved at the exception of the D300 in HAd11K which is replaced by V300 in HAd7K. In particular, aspartic acid 265 (D265) in HAdV-7 knob is conserved in HAdV-11 knob. The region adjacent to D265 in HAd11K is highly conserved except a neutral substitution (R266 in the HAd11K; H266 in the HAd7K). We generated mutant HAd11K D265A. This mutant was subjected to BLI analyses, showing an inability to interact with rDSG2, which clearly indicates that as for other adenoviruses interacting with DSG2 receptor, D265 is a key residue for stabilizing the complex with this receptor since its mutation drastically reduces the interaction to DSG2 (KD = 7.218 × 10^−5^) as shown by the BLI experiment (Fig. 5F). HAd11K has earlier been reported to compete for the same cellular receptor as whole HAdV-5/11 virus. To assess whether D265 may affect virus cell binding, cells were incubated with a HAd11K D265A mutant before the addition of HAdV-5/11 virus. The presence of mutated fiber, which can bind to CD46 but can no longer interact with DSG2, only partially abolishes the binding of the whole virus to the cell (Fig. 5G). These results could be in favor of an opportunistic behavior of the virus which uses the DSG2 receptor when CD46 is blocked or absent.

### HAd11K binds simultaneously to rCD46 and rDSG2 under certain conditions

**Fig 6:**
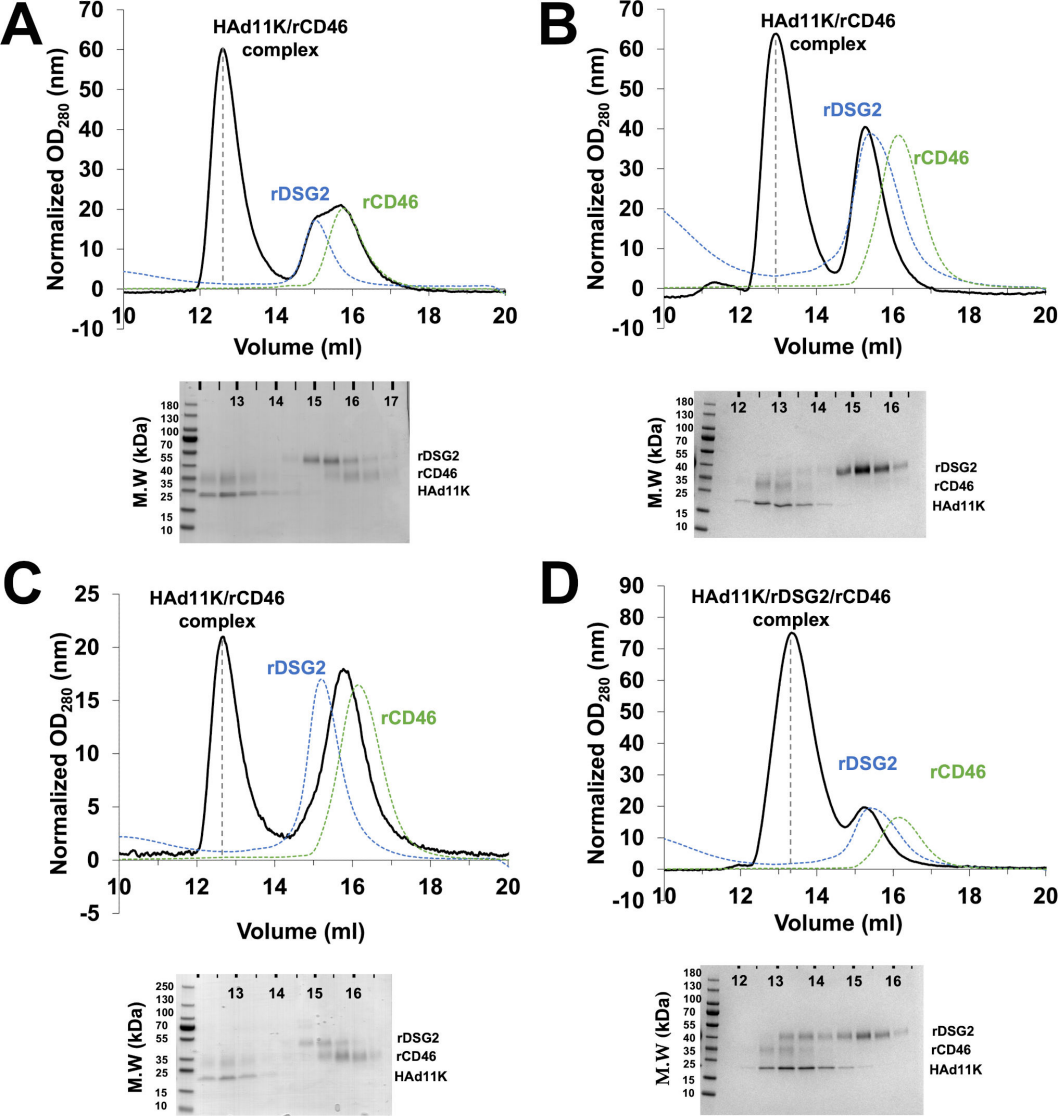
Analysis of complex formation between HAd11K and its receptors by size-exclusion chromatography. Mixtures of HAd11K with rDSG2 and rCD46 were examined in four different configurations: (**A**) HAd11K, rDSG2, and rCD46 were co-incubated at the same time; (**B**) pre-formed HAd11K/rDSG2 complex was co-incubated with rCD46; (**C**) pre-formed HAd11K/rCD46 complex was co-incubated with rDSG2; and (**D**) HAd11K, excess of rDSG2 (molar ratio of rDSG2 to rCD46, 3:1) and rCD46 were co-incubated at the same time. Eluted fractions corresponding to black chromatograms were analyzed by SDS-PAGE, followed by Coomassie blue staining. Dashed lines indicate elution of rDSG2 (blue) and rCD46 (green) injected alone on the same column.

To begin to address the question of simultaneous binding of both DSG2 and CD46 on HAd11K, the same approach was used to form the HAd11K/rDSG2 complex. rDSG2, rCD46, and HAd11K were co-incubated at various molar ratios overnight at 4°C prior to gel filtration, and eluted fractions were analyzed on a SDS-PAGE gel. When rCD46 and rDSG2 were simultaneously mixed together with HAd11K at a 1:1:1 molar ratio (Fig. 6A), rCD46 co-eluted with HAd11K at a higher elution volume (12.6 mL) with an excess eluted around 16 mL. Unexpectedly, the whole rDSG2 is detected only in its free form in the last eluted fractions (15 mL). To ascertain whether, when bound to one of its receptor, HAd11K maintained the ability to physically interact with the other, soluble rCD46 was added to a preformed complex made of rDSG2 and HAd11K (Fig. 6B) or soluble rDSG2 was added to a preformed complex made of rCD46 and HAd11K (Fig. 6C). The resulting complexes were then analyzed by gel filtration. In both configurations, only HAd11K/rCD46 complex appears on the elution profiles. This indicates not only that in the presence of rCD46, rDSG2 fails to bind to the HAd11K/rCD46 preformed complex but also that addition of rCD46 can displace rDSG2 from the preformed HAd11K/rDSG2 complex. In order to limit this displacement, excess of soluble rDSG2 (molar ratio of rDSG2 to rCD46, 3:1) was mixed with rCD46 and HAd11K (Fig. 6D). Interestingly, the elution profile shows the presence of a super-complex of HAd11K with its two receptors. The fraction corresponding to the complex was recovered and proceeded to cryo-EM.

### Structure determination of the HAd11 knob in complex with rDSG2 and rCD46

**Fig 7:**
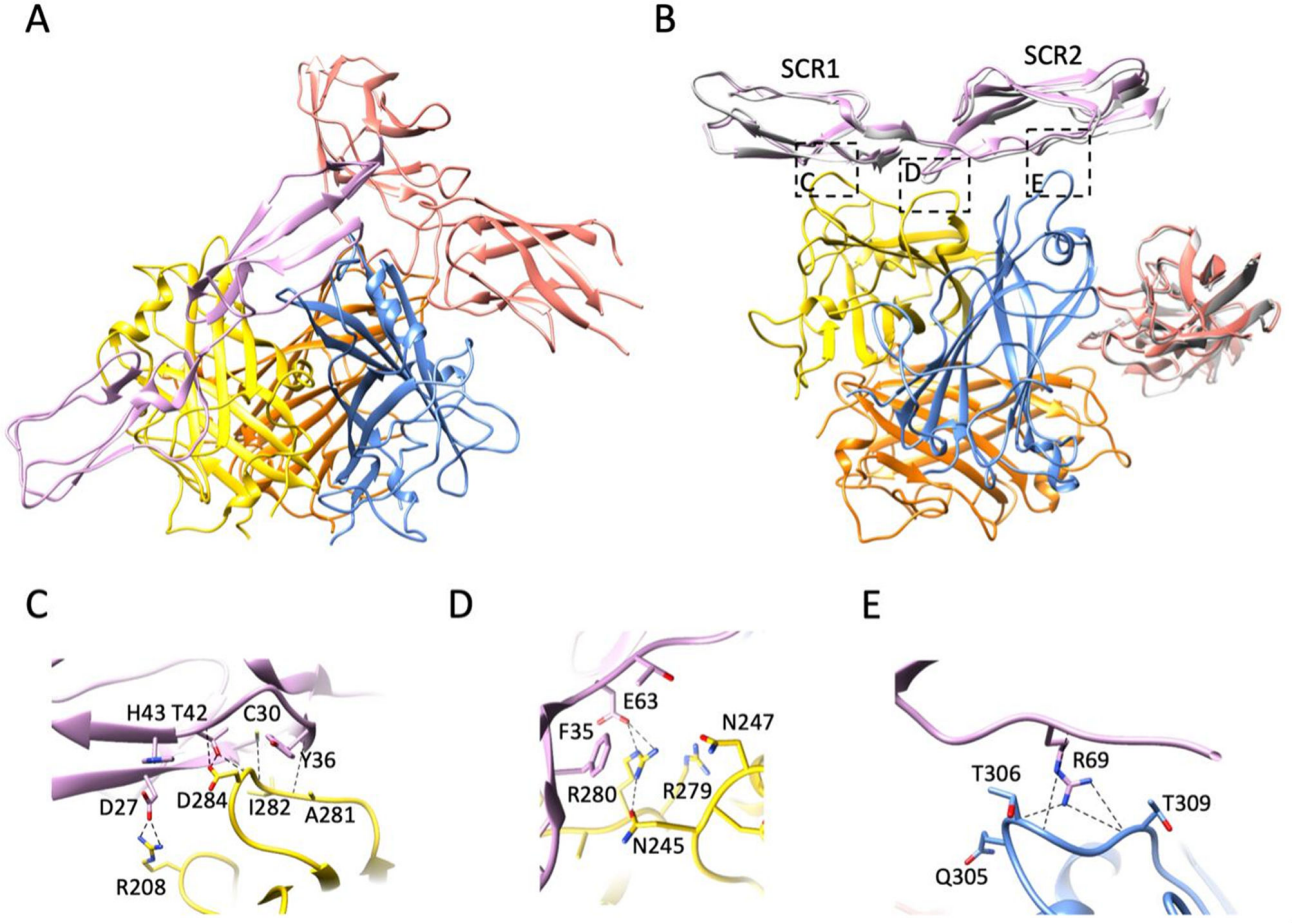
(A) Ribbon representation of a side view of the HAd11K trimer (orange, yellow, and blue subunits) in complex with rDSG2 (salmon) and rCD46 (pink). (B) Overview of the three interaction sites of rCD46 on two different HAd11K monomers using the same color code as in A. Superimposed onto the rDSG2 molecule (salmon) is a ribbon representation of an rDSG2 molecule (gray), which was determined in complex with the HAd11 knob (see Fig. 5). Superimposed onto the rCD46 molecule (pink) is a ribbon representation of an rCD46 molecule (gray), which was determined in complex with the HAd11 knob by X-ray crystallography (PDB ID:2O39) (17). (C–E) zoomed views on the three contact zones between rCD46 and HAd11K highlighted in (**B**). Putative hydrogen bonds and salt bridges are shown in dotted black lines.

A large data set of HAd11K/rDSG2/rCD46 was acquired by cryo-EM, and the image analysis shows that there was a mixture of different complexes in solution even though the molecular ratio of rDSG2 to rCD46 was optimized to produce a ternary complex. Indeed, populations composed of rDSG2 and rCD46 alone were detected along with a “super complex” of HAd11K interacting with one rDSG2 and one rCD46 molecule (Fig. 7A and Fig. S2). As both rDSG2 and rCD46 bind with two different monomers of HAd11K, they have one interacting monomer in common. But as rDSG2 binds to the apex of the knob and rCD46 the periphery of it, they do not prevent each other from simultaneously attached to the trimeric knob. In the ternary complex, rDSG2 binds to HAd11K in a nearly identical way as when rCD46 is not included (Fig. 7B; Table S2), and the details of the interaction are, therefore, not recapitulated here. In the super-complex, rCD46 binds to two different HAd11K monomers at the periphery of the knob (Fig. 7B) in a very similar way as it has been reported in a previous crystal structure (17) (Table S2). Indeed, the HI and DG loops of one monomer make extensive contacts with the SCR1 (Fig. 7C and Fig. S1) and the SCR1-SCR2 interface (Fig. 7D and Fig. S1) and the IJ loop of the neighboring monomer interacts with the base of SCR2 (Fig. 7E and Fig. S1). Key residues for the rCD46/HAd11K interaction identified in the crystal structure are similarly important in the cryoEM structure such as the salt bridges and hydrogen bonds between H43 of rCD46/D284 of HAd11K (Fig. 7D and Fig. S1) and E63 of rCD46/R280 of HAd11K (Fig. 7E and Fig. S1).

## DISCUSSION

In this study, we deciphered the respective role of DSG2 and CD46 in HAdV-11 entry. Studying the tropism of adenoviruses is critical for developing new vectors. Indeed, the use of oncolytic viruses (OVs) represents a promising therapeutic approach against cancer. Oncolytic adenoviruses (OAds) are well-studied due to their powerful capacity in oncolysis and immune response stimulation, and oncorine was the first approved OAd in China (22). OAd infects tumor cells through the interaction of adenoviral fiber knob with receptors on the cell surface (23, 24). HAdV-5 was the most commonly used serotype for the design of conventional OAds and binds primarily to the coxsackievirus and adenovirus receptor (CAR) (25, 26). Since CAR is absent or poorly expressed in many cancer cells, alternative strategies using non-CAR binding HAdVs were developed. To improve tumor tropism, chimeric group B oncolytic adenoviruses, like HAdV-5/3 (HAdV-5 pseudotypes with the HAdV-3 fiber), fully serotype 3 (27) or chimeric HAdV-11p/3 (Enadenotucirev) viruses were investigated because of their ability to use either CD46 (2) and/or desmoglein-2 (DSG2) (3) as their primary receptors. Of note, Enadenotucirev is a chimeric HAdV-11p/HAdV-3 virus, but the capsid is fully made of HAdV-11 capsomers (15, 28) that dictate its tropism. Deciphering the respective role played by CD46 and DSG2 in HAdV-11 entry is then highly desirable. We first performed kinetics studies to compare affinity of interaction between the two receptors (Fig. 1). Since previous experiments indicated that the binding of fiber knob to immobilized CD46 could not be modeled by a simple 1:1 interaction scheme (29), we chose to use fiber knob as the ligand. This configuration allows us to measure the binding of both CD46 and DSG2 molecule without being influenced by potential avidity effects. The measured *K*_D_ values for DSG2 to HAd11K were about 12-fold higher than for CD46 with 2900 nM and 240 nM, respectively. The affinity appeared to be weaker for the two receptors than those specified in other studies (30), but it should be noted that the experimental settings were quite different. Indeed, in this study, we used monomeric recombinant receptors DSG2 (EC2-EC3) or CD46 (SCR1-SCR2). Whereas in the previous work, the affinity constants were determined between DSG2 and the fiber knob using a dimeric recombinant DSG2 consisting of the three extracellular domains (EC1-EC2-EC3) fused to a dimeric Fc fragment (19) resulting in an avidity phenomenon and, in turn, in a better apparent *K*_D_.

The better affinity of HAd11K to CD46 raised the question of the role played by DSG2 in viral entry. To address this point, a CHO-DSG2 cell line was generated. Incubation of HAdV-5/11 encoding GFP (HAdV-5/11-GFP) clearly demonstrated that DSG2 is sufficient to mediate viral entry (Fig. 3). It remains to determine whether the density of receptor at the cell surface is critical as previously observed for HAdV-3 interaction with CD46 which only occurred at high density contrary to its interaction with DSG2 (31). To go further in the mechanism understanding, we used multiple approaches to competitively inhibit the attachment of HAd11K to CD46 or HAdV-5/11 to host cell (Fig. 2). Whereas soluble rCD46 abolished fiber knob interaction with immobilized CD46, soluble rDSG2 only partially reduced the attachment. Similarly, rCD46 significantly inhibited HAdV-5/11 entry in cell unlike rDSG2. However, that inhibition by the addition of soluble CD46 receptor was not complete, suggesting that even if CD46 is the main receptor, virus entry may be, in part, mediated by other receptors or mechanisms. A structural study has then been performed by Cryo-EM with HAd11K in complex with rDSG2 alone (Fig. 5). No structure was available for the interaction of HAd11K in complex with rDSG2. As reported for the other two serotypes, −3 and −7, the structure shows that EC2 and EC3 ectodomains bind at the top of the trimeric knob and that D265 mediates central contacts with DSG2 (19, 21). Interestingly, the critical D265 residue interacting with the EC3 domain of DSG2 is also seen in HAd11K confirming the importance of this pocket for all DSG2-interacting serotypes. We, therefore, produced a D265A mutant of HAd11K and subjected to BLI experiments, clearly indicated that D265 is, indeed, a key residue for stabilizing the complex with DSG2 (Fig. S2).

To go beyond, we tried to produce Ad11K/receptor “super-complexes” in which CD46 and DSG2 would coexist. The multiple attempts to obtain such a complex showed that the attachment of CD46 to the fiber knob takes precedence over that of DSG2 (Fig. 6). Once formed, the HAd11K/CD46 complex does not allow DSG2 binding. Conversely, the HAd11K/DSG2 complex can be dissociated by the addition of CD46. By playing with the stoichiometry of the two receptors, we successfully obtained a “super-complex” composed of the HAd11K trimeric fiber knob with only one molecule of DSG2 and only one molecule of CD46 (Fig. 7).

This shows for the first time that a physical super-complex can be generated at least *in vitro*. However, the presence of such complex at the cell surface remains to be proved to answer the question of whether the two receptors are used at once or subsequently during the viral spreading through the tissue. Moreover, as the virus has 12 fibers, it cannot be excluded that if the concomitant interaction with the 2 receptors does not take place on the same fiber, and it cannot take place on two independent fibers of the virion, making the study of the tropism of this virus more complex. The location of these receptors at the cell surface is, thus, critical. CD46 is a transmembrane glycoprotein, widely expressed on most cells and generally located at the basolateral and apical cell surface (32, 33). Conversely, DSG2 participates in the formation of desmosomes and is a component of tight intercellular junctions (34). It is now accepted that many species B and D HAdVs can use more than one cellular receptor to achieve infection. For instance, DSG-2 functions as a major attachment receptor for HAdV-3, whereas CD46 exerts a minor contribution to virus attachment (10). A more recent study also demonstrated that both DSG2 and CD46 are able to mediate HAdV-55 infection, but DSG2 plays the major roles (35).

Surprisingly, this property does not seem to make the serotype 11 more infectious and virulent, as epidemics related to this serotype remain rare, as evidenced by the low seroprevalence in humans (7). However, the flexibility in the usage of these two receptors could explain, in part, the clinical efficacy of enadenotucirev (8, 28, 36). Some matrix metalloproteinases have been shown to mediate the ectodomain shedding of DSG2 from the cells. Our data suggest that exposure to circulating DSG2 ectodomains should not compromise HAdV-11 attachment to the host cell. Indeed, even if cleaved DSG2 is bound to the fiber, this does not prevent interaction with CD46. This is particularly important for oncolytic vector given that shedding of desmosomal cadherin ectodomains by cellular proteases has been reported during cancer progression and metastasis.

Our work deciphering the usage of two independent receptors by HAdV-11 from a biochemical and structural point of view is one more piece added to the understanding of adenovirus tropism. Based on our data, it is now possible to design new adenoviral vectors partially detargeted for one or the other of these two receptors and investigate their spreading in tumors expressing different levels of either CD46 or DSG2.

## MATERIALS AND METHODS

### Viruses

HAd5/11-GFP was kindly provided by Pr André Lieber (University of Washington). Virus was amplified on HEK293 cell and purified by cesium chloride banding as described in Kanegae et al. (37). Virus was desalted on P10 column with PBS-10% glycerol. The titer in physical particles (viral particles; vp) was determined by absorbance measurement at 260 nm (*A*_260_) of 1 mL samples of SDS-denatured virions (0.1% SDS for 1 min at 56°C) in a 1-cm-path-length cuvette, using the respective formula: *A*_260_ of 1.0 = 1.1610^12^ vp/mL for HAdV-5/11 (genomic DNA = 36 kbp). All the experiments were performed with the same batch.

### Cell lines

HeLa, Chinese hamster ovary CHO-K1, and human embryonal kidney HEK293 F cells were kept in our lab. Stable CHO cell line expressing hDSG2 has been developed on our request by Innoprot (Spain). HEK293 F cells were maintained in FreeStyle 293 Expression medium (Gibco, Thermo Fisher Scientific) in vented Erlenmeyer flasks (Corning) at 120 rpm, in a 37°C, 5% CO_2_, humidified incubator. HeLa cells were cultured at 37°C under 5% CO_2_ atmosphere in RPMI 1640 medium supplemented with 10% fetal calf serum (FCS), penicillin, and streptomycin (50 µg/mL). After trypsinization, 1.10^5^ cells per well were distributed on a 4-well plates (Nunclon, delta surface) and cultured overnight in 500 µL of complete medium before the experiments. CHO-K1 were maintained in DMEM medium supplemented with 10% FCS, penicillin, and streptomycin (50 µg/mL). 4-well plates were seeded with 1.10^5^ cells and infected with at the indicated M.O.I. after overnight incubation. Immunofluorescence pictures were taken 24H post infection on ZOE Fluorescent Cell Imager (Bio-Rad) using the green and brightfield channels.

CHO-DSG2 were grown at 37°C under 5% CO_2_, in Dulbecco’s modified Eagle’s medium-high glucose (Sigma-Aldrich) supplemented with 10% FBS, 10 µg/mL Puromycin (Sigma-Aldrich), and 1 × MEM non-essential-amino-acid solution (Sigma-Aldrich) and prepared for infection as described above for CHO-K1. Images were taken on ZOE as decribed above.

### Prokaryotic expression plasmid

HAdV-11 fiber protein (residues D128 to D325, Swiss-Prot: P35774) was chemically synthetized by GenScript and cloned in pETDuet-1 plasmid with a hexahistidine-tag in N-terminus. A single colony was inoculated into LB medium containing ampicillin (50 µg/mL) and chloramphenicol (34 µg/mL) and grown overnight at 37°C. The culture was subsequently diluted to 1/100th into LB medium supplemented with ampicillin and chloramphenicol and incubated at 37°C until an OD of 0.4–0.6 was reached. To induce protein expression, IPTG was added to a final concentration of 1 mM, and the culture was incubated at 18°C. Bacteria were harvested by centrifugation 18 h after induction. Pellets were resuspended in lysis buffer [500 mM NaCl, 3 mM CaCl_2_, 10 mM Imidazole, 20 mM Tris-Cl, pH 8.0 and cOmplete EDTA-free Protease Inhibitor Cocktail (Roche Diagnostics)] and lysed by sonication. Supernatant was clarified by centrifugation at 39,000*g* for 30 min at 4°C.

### Mammalian expression plasmid

Plasmid expressing rDSG2 was constructed by inserting the coding sequence of the EC2-EC3 domains (residues V149-I386, Swiss-Prot: Q14126.2) using the restriction sites AgeI/KpnI in pHL-sec plasmid (Addgene plasmid # 99845) as described in reference (38). It contains a Kozak sequence, a secretion signal sequence, and a C-terminal hexahistidine-tag. For plasmid expressing rCD46, the coding sequence for the two first extracellular domains of hCD46 (residues C35-V160, Swiss-Prot: P15529) was synthetized by GenScript and cloned into the same expression vector pHL-sec.

### Transient transfection

One day before transfection, HEK293 F were seeded in fresh culture medium at 0.6 × 10^6^ cells/mL. Cells were transiently transfected when the density reached 1 × 10^6^ cells/mL. DNA was added to Lipofectamine 2000 (LifeTechnologies) in a 1:2.5 ratio, incubated at room temperature for 25 min, and added to the cells according to the manufacturer’s manual. Supernatants were recovered 3 days post transfection by centrifugation at 1,500 × *g*, 10 min at 4°C. cOmplete EDTA-free Protease Inhibitor Cocktail was added to the cleared supernatant prior to storage at −20°C.

### Purification of His-tagged proteins

Recombinant His-tagged proteins were purified as described previously (20). Briefly, clarified supernatants were loaded onto 1 mL His GraviTrap prepacked columns (Cytiva). Eluted proteins with high concentrations of imidazole were concentrated and injected onto a Superdex 200 Increase 10/300 Gl column (Cytiva) equilibrated with gel filtration buffer (150 mM NaCl, 3 mM CaCl_2_, 10 mM Tris-Cl, pH 8.0). Fractions corresponding to the major peak were analyzed by SDS-PAGE, and proteins were concentrated to 1–2 mg/mL.

### Bio-layer interferometry

Measurements were performed on an Octet N1 BLI Technology instrument (Sartorius). Prior to experiments, Amine Reactive 2nd Generation (AR2G) biosensors (Sartorius) were hydrated in water for at least 10 min. His-tagged HAd11K WT (wild type) or mutants (D265A) were diluted to 100 µg/mL in 10 mM sodium acetate, pH 4.0, and immobilized on the biosensors using the amine coupling chemistry according to the manufacturer instructions. Initial baseline was then recorded for 30 s. The association and dissociation sensorgrams with rDSG2 or rCD46 were monitored in 10 mM HEPES, 145 mM NaCl, 0.005% surfactant P20, pH 7.4 (HBS-P) (Cytiva) with 3 mM CaCl_2_ at concentrations ranging from 0 to 10 µM. Equilibrium dissociation constant (*K*_D_) and association (*k*_on_) and dissociation (*k*_off_) rate constants were calculated from the BLI data using the global fitting method provided in data analysis software BLItz Pro version 1.2.1.3. For competition assays, Fc CD46 was captured on protein A biosensors (Sartorius) in HBS-P buffer with 3 mM CaCl_2_. HAd11K was incubated for 4H at 4°C in the presence of rCD46 or rDSG2 before injection.

#### Virus infection assays

HAd5/11-GFP (2.5 10^8^ vp) was incubated with either soluble rCD46 (SCR1-2 domains) or soluble rDSG2 (EC2-3 domains) or both together at the indicated concentration, for 3H at room temperature in 250 µL of medium without serum. HeLa cells cultured in 4-well plates were washed twice with 500 µL of cold medium without serum before the addition of the virus-competitor mix pre-chilled at 4°C. After 1H of contact at 4°C, samples were withdrawn and the wells were washed twice with 500 µL of cold medium without serum. Five hundred microliters of complete medium was then added, and cells were incubated for 24H at 37°C. For immune-fluorescence studies, plates were imaged by ZOE (Bio-Rad) using the brightfield and GFP channels and images were merged.

### Flow cytometry analysis

For FACS analysis, cells were detached by 100 µL of trypsin for 5 min at 37°C, 400 µL of PBS was added, and cell suspensions were spin for 1 min on table top centrifuge. Pellets were resuspended in 500 µL of PBS. FACS analysis was performed on MACSQuant-VYB instrument (Miltenyi Biotec). Analysis of GFP-positive cells was performed on MACSQuant software using non-infected cells to determine the threshold of GFP intensity representative of infection, on a total of 2.10^4^ single cells. Results were normalized using the condition of HAd5/11 without competitor as 100% of infection.

### Cryo-electron microscopy of HAd11k in complex with rDSG2

#### Sample preparation and image acquisition

3.5 µL of sample was applied to 1.2/1.3 Quantifoil holey carbon grids (Quantifoil Micro Tools GmbH, Germany) and plunged frozen in liquid ethane with a Vitrobot Mark IV (Thermo Fisher Scientific) (6 s blot time, blot force 0). The sample was observed at the beamline CM01 of the ESRF (Grenoble, France) (39) with a Titan Krios G3 (Thermo Fischer Scientific) at 300 kV equipped with an energy filter (Bioquantum LS/967, Gatan Inc, USA) (slit width of 20 eV). Five thousand three hundred thirty-eight movies were recorded automatically on a K2 summit direct detector (Gatan Inc., USA) in super resolution mode with SerialEM (40). Movies were recorded for a total exposure of 6 s and 150 ms per frame resulting in 40 frame’s movies with a total dose of ~55 e−/Å^2^. The magnification was 215,000 × (0.325 Å/pixel at the camera level). The defocus of the images varied between −1.0 and −2.5 µm.

#### Image analysis

The movies were first drift-corrected and binned two times by Fourier cropping with motioncor2 (41). The remaining image processing was done in RELION 3.08 (42) and 3.1 (43). CTF estimation was done with GCTF (44). An initial set of particles (box size of 160 pixels, sampling of 1.3 Å/pixel) was obtained by auto-picking with a gaussian blob. After a 1st 2D classification, the particles belonging to the best-looking 2D class averages were used to do a template-based autopicking. Following another 2D classification, a first 3D classification (C1, no mask, 10 classes) was done using as a starting model a model of HAd3K in complex with one rDSG2 (21) to further eliminate bad particles. A first 3D reconstruction with two rDSG2 was then obtained which was then used in two successive rounds of 3D classifications (C1, no mask, 5 and 3 classes). At that stage of the image analysis, two classes of particles consisting of HAd11K in complex with either one or two rDSG2 were obtained. The 3D reconstructions for these two classes were then further improved independently by 3D classification and 3D refinement. Particle polishing and CTF refinement (anisotropic magnification, beam tilt, trefoil, fourth-order aberrations, defocus refinement per particle, and astigmatism refinement per micrograph) were then performed for both classes followed by a last 3D refinement. The final 3D reconstructions were calculated with 105,169 and 127,522 particles for HAd11K with one or two rDSG2, respectively. Resolutions of 3.5 and 3.3 Å were determined by Fourier Shell Correlation (FSC) at 0.143 for HAd11K with one or two rDSG2, respectively. The final 3D reconstructions were sharpened with deepEMhancer (45).

### Cryo-electron microscopy of HAd11k in complex with rDSG2 and rCD46

#### Sample preparation and image acquisition

3.5 µL of sample was applied to 1.2/1.3 Quantifoil holey carbon grids (Quantifoil Micro Tools GmbH, Germany) and plunged frozen in liquid ethane with a Vitrobot Mark IV (Thermo Fisher Scientific) (6–7 s blot time, blot force 0). The sample was observed at the beamline CM01 of the ESRF (Grenoble, France) (39) with a Titan Krios G3 (Thermo Fischer Scientific) at 300 kV equipped with an energy filter (Bioquantum LS/967, Gatan Inc, USA) (slit width of 20 eV). Eleven thousand seven hundred eighty seven movies were recorded automatically on two different grids with a K3 direct detector (Gatan Inc., USA) in super resolution mode with EPU (Thermo Fischer Scientific). Movies were recorded for a total exposure time of 2.3 s with 60 frames per movie and a total dose of ~58 e−/Å^2^. The magnification was 105,000 × (0.42 Å/pixel at the camera level). The defocus of the images varied between −1.0 and −2.5 µm.

#### 3D reconstruction

For both data sets, the movies were first drift-corrected and binned two times by Fourier cropping with motioncor2 (41). The remaining image processing was done in RELION 4.0 (46). CTF estimation was done with GCTF (44). The image analysis was then initially focused on the first data set. An initial set of particles (box size of 100 pixels, sampling of 2.1 Å/pixel) was obtained by auto-picking with a gaussian blob. After a 1st 2D classification, the particles belonging to the best looking 2D class averages were used to do a template-based autopicking. Following two more 2D classifications, an initial model displaying mostly HAd11K with 1 rCD46 was obtained *ab initio* with RELION and used for a first 3D refinement. Two successive 3D classifications (with 5 and 3 classes, respectively) allowed the isolation of a well-defined population of particles consisting of HAd11K with 1 rCD46 and 1 rDSG2. A first 3D map at 4.4 Å resolution was then obtained for HAd11K with 1 rCD46 and 1 rDSG2. In an attempt to increase the quantity of particles for the first grid, the 4.4 Å 3D map of HAd11K with 1 rCD46 and 1 rDSG2 was used as a reference for further 3D classifications on the particles obtained after the 2D classification steps. To do so, four consecutive 3D classifications (with 5, 10, 7, and 3 classes, respectively) were done which allowed the identification of a set of 114,694 particles of HAd11K with 1 rCD46 and 1 rDSG2 for the first grid.

For the second grid, 2D class averages from the first grid were used as template for picking automatically the micrographs. Following two successive 2D classifications to eliminate the wrongly picked particles, the 3D map of HAd11K with 1 rCD46 and 1 rDSG2 from the first grid was used as template for three consecutive 3D classifications (with 5, 5, and 3 classes, respectively) which allowed the identification of clean population of HAd11K with 1 rCD46 and 1 rDSG2 for the second grid consisting of 65,816 particles.

The two set of particles from the two different grids were combined and, following CTF refinement (anisotropic magnification, beam tilt, trefoil, fourth-order aberrations, defocus refinement per particle, and astigmatism refinement per micrograph) and particle polishing, the final 3D reconstruction for HAd11K with 1 rCD46 and 1 rDSG2 was calculated from 180,510 particles. A resolution of 3.2 Å was determined by FSC at 0.143. The final 3D reconstruction was sharpened with deepEMhancer (45).

#### Model building and refinement

The crystal structures of the HAd11K/CD46 [PDB 3O39 (17)] and the DSG2 module from a previous work on HAd7K (19) were rigid-body fitted inside the cryo-EM density maps of either HAd11K in complex with rDSG2 alone or rDSG2 and rCD46 in CHIMERA (47). The atomic coordinates were then refined with ROSETTA (48) and PHENIX (49). The refined atomic models were visually checked and adjusted (if necessary) in COOT (50). The final atomic models were validated with MOLPROBITY (51).

Analysis of the interaction between the rDSG2 or rCD46 with HAd11K was done with PISA (52) [“Protein interfaces, surfaces, and assemblies” service PISA at the European Bioinformatics Institute (http://www.ebi.ac.uk/pdbe/prot_int/pistart.html)]. Any putative interaction identified with PISA for which the distance between atoms was more than 3.5 Å for hydrogen bond and 4 Å for salt bridge was discarded. The figures were prepared with CHIMERA. The data collection and model statistics are summarized in Table S1 and S2. Structural biology applications used in this project were compiled and configured by SBGrid (53).

## Data Availability

The atomic coordinates and the cryo-EM maps have been deposited in the PDB and EMDB, respectively. Atomic coordinates for HAd11k with one rDSG2, HAd11k with two rDSG2, and HAd11k with rDSG2 and rCD46 have PDB accession codes 8QJY, 8QJX, and 8QK3, respectively, while the cryo-EM maps have EMDB accession codes EMD-18454, EMD-18453, and EMD-18455, respectively.

## SUPPLEMENTAL MATERIAL

Fig. S1 jvi.00910-23-s0001.pdf Cryo-EM field of view of HAd11K in complex with rDSG2.

Fig. S2 jvi.00910-23-s0002.pdf Cryo-EM field of view of HAd11K in complex with rDSG2 and rCD46.

Table S1 jvi.00910-23-s0003.pdf Root Mean Square Deviation (RMSD) between some of the available rDSG2 and rCD46 structures.

Table S2 jvi.00910-23-s0004.pdf Summary of data collection and atomic model statistics.

ASM does not own the copyrights to Supplemental Material that may be linked to, or accessed through, an article. The authors have granted ASM a non-exclusive, world-wide license to publish the Supplemental Material files. Please contact the corresponding author directly for reuse.
