# Supplementary material for: Toward the understanding of DSG2 and CD46 interaction with HAdV-11 fiber, a super-complex analysis
Source: J Virol. 2023 Nov 3;97(11):e00910-23. doi: 10.1128/jvi.00910-23 (PMC10688334; doi:10.1128/jvi.00910-23)
Supplement: Fig. S1 — Cryo-EM field of view of HAd11K in complex with rDSG2. [file jvi.00910-23-s0001.pdf]

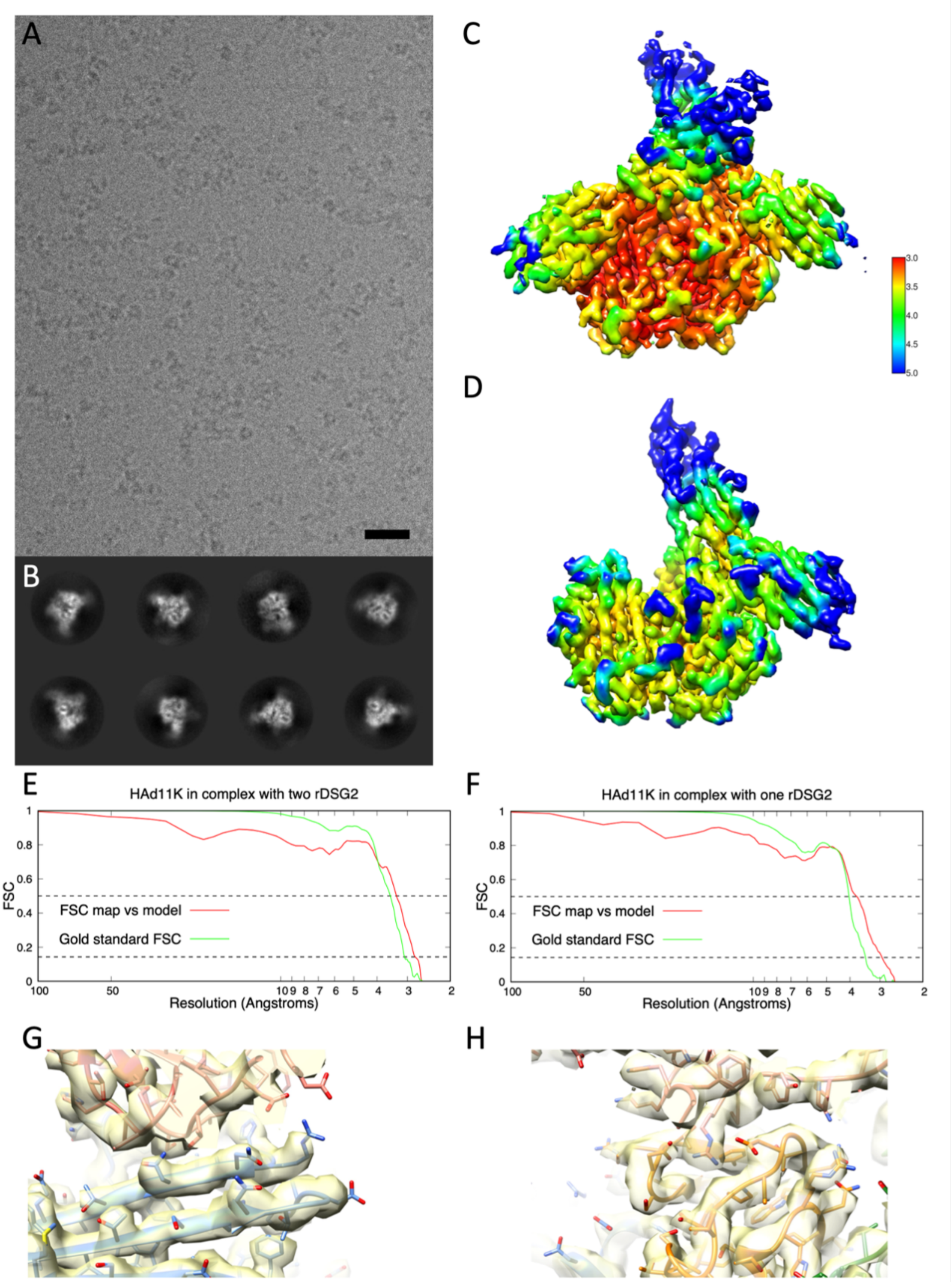

Figure S1*: A - Cryo-EM field of view of HAd11K in complex with rDSG2. Scale bar represents 20 nm. B- Representative 2D class averages of HAd11K in complex with rDSG2. C &D - Local resolution maps of the 3D reconstructions obtained for HAd11K in complex with either one (C)*

or two (D) rDSG2s. E & F- Fourier Shell Correlation (FSC) curves for HAd11K in complex with two (E) or one (F) rDSG2. For each plot, the gold standard FSC between two independent 3D reconstructions is shown in red while the FSC curve between the cryo-EM coulomb potential map and the corresponding refined atomic model is in green. The two dotted horizontal lines represent FSC=0.143 and 0.5 which are used as cutoffs to determine the resolutions for the "Gold standard FSC" and the "FSC map vs model" respectively. G & H- Illustrations of the quality of the obtained 3D reconstruction and atomic model for HAd11K in complex with two rDSG2. The coulomb potential map from cryo-EM is in transparent yellow. Panel G is the same view as Figure 5D and is centered on the interaction of the EC2 module of rDSG2 (salmon) with a monomer of the HAd11K (blue). Panel H is the same view as Figure 5E and is centered on the interaction of the EC3 module of rDSG2 (salmon) with a monomer of the HAd11K (orange).
