# Supplementary material for: Toward the understanding of DSG2 and CD46 interaction with HAdV-11 fiber, a super-complex analysis
Source: J Virol. 2023 Nov 3;97(11):e00910-23. doi: 10.1128/jvi.00910-23 (PMC10688334; doi:10.1128/jvi.00910-23)
Supplement: Fig. S2 — Cryo-EM field of view of HAd11K in complex with rDSG2 and rCD46. [file jvi.00910-23-s0002.pdf]

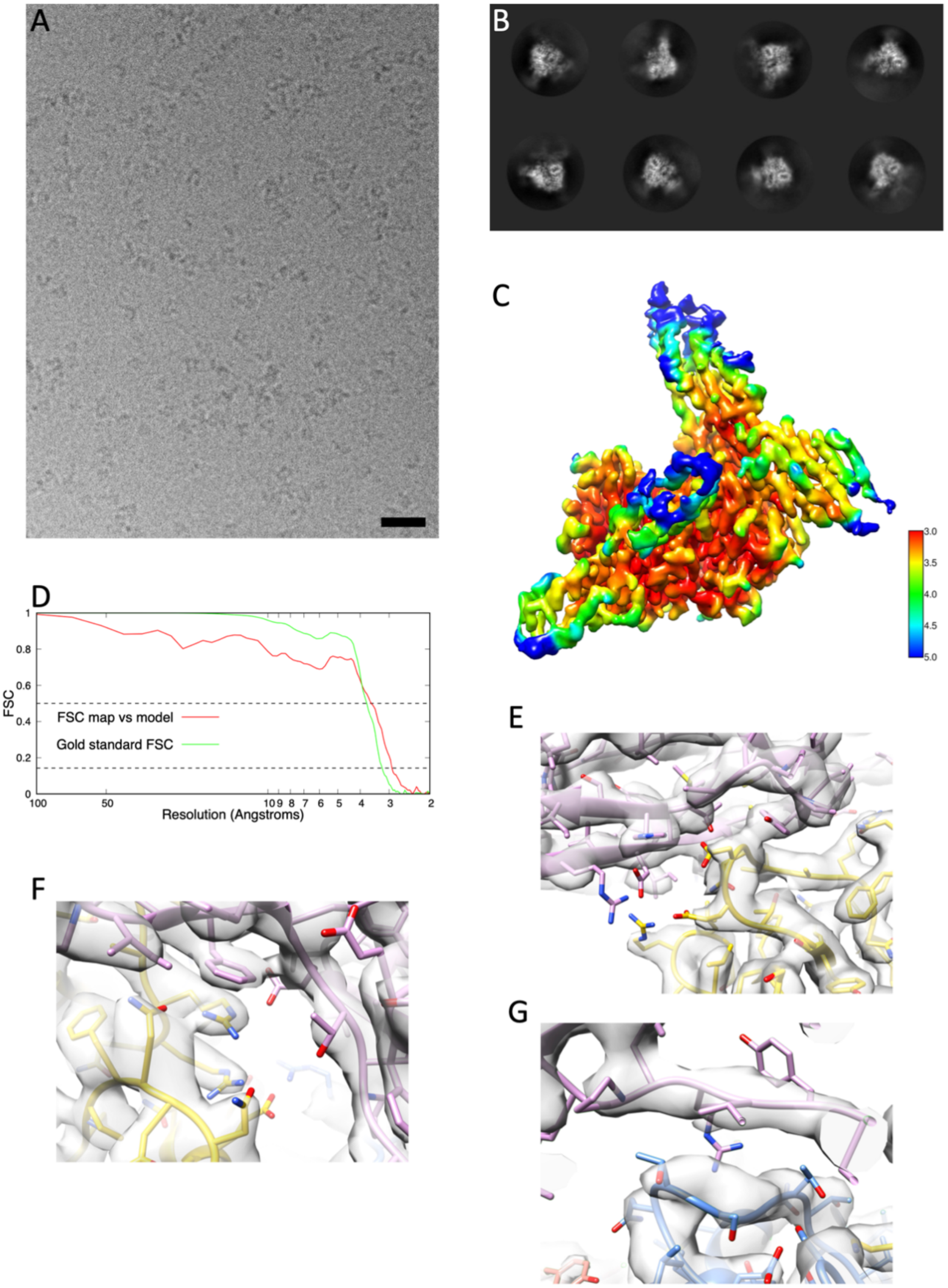

Figure S2*: A - Cryo-EM field of view of HAd11K in complex with rDSG2 and rCD46. Scale bar represents 20 nm. B- Representative 2D class averages of HAd11K in complex with rDSG2 and rCD46. C - Local resolution map of the 3D reconstructions*

obtained for HAd11K in complex with rDG2 and rCD46. D- Fourier Shell Correlation (FSC) curves for HAd11K in complex with rDSG2 and rCD46. The gold standard FSC between two independent 3D reconstructions is shown in red while the FSC curve between the cryo-EM coulomb potential map and the corresponding refined atomic model is in green. The two dotted horizontal lines represent FSC=0.143 and 0.5 which are used as cutoffs to determine the resolutions for the "Gold standard FSC" and the "FSC map vs model" respectively. E to G- Illustrations of the quality of the obtained 3D reconstruction and atomic model for HAd11K in complex with rDSG2 and rCD46. The coulomb potential map from cryo-EM is in transparent grey. Panel E is the same view as Figure 7C and is centered on the interaction of the SCR1 module of rCD46 (pink) with a monomer of the HAd11K (yellow). Panel F is the same view as Figure 7D and is centered on the interaction occurring between the SCR1-SCR2 interface of CD46 (pink) with a monomer of the HAd11K (yellow). Panel G is the same view as Figure 7E and is centered on the interaction of the SCR2 module of rCD46 (pink) with a monomer of the HAd11K (blue).
