# Supplementary material for: Toward the understanding of DSG2 and CD46 interaction with HAdV-11 fiber, a super-complex analysis
Source: J Virol. 2023 Nov 3;97(11):e00910-23. doi: 10.1128/jvi.00910-23 (PMC10688334; doi:10.1128/jvi.00910-23)
Supplement: Table S1 — Root Mean Square Deviation (RMSD) between some of the available rDSG2 and rCD46 structures. [file jvi.00910-23-s0003.pdf]

Table S1: Root Mean Square Deviation (RMSD) between some of the available rDSG2 and rCD46 structures

**Between**		**RMSD (Å)**
rDSG2 in cryo-EM map of HAd11K/one rDSG2	rDSG2 in cryo-EM map of HAd11K/two rDSG2	1.15
First rDSG2 in cryo-EM map of HAd11K/two rDSG2	Second rDSG2 in cryo-EM map of HAd11K/two rDSG2	1.23
rDSG2 in cryo-EM map of HAd11K/ rDSG2/rCD46	Second rDSG2 in cryo-EM map of HAd11K/two rDSG2	1.22
rCD46 in cryo-EM map of HAd11K/ rDSG2/rCD46	rCD46 in crystal structure pdbID: 2O39	1.31
HAd11K trimer in cryo-EM map of HAd11K/ rDSG2/rCD46	HAd11K trimer in crystal structure pdbID: 2O39	0.54
