# Supplementary material for: Toward the understanding of DSG2 and CD46 interaction with HAdV-11 fiber, a super-complex analysis
Source: J Virol. 2023 Nov 3;97(11):e00910-23. doi: 10.1128/jvi.00910-23 (PMC10688334; doi:10.1128/jvi.00910-23)
Supplement: Table S2 — Summary of data collection and atomic model statistics. [file jvi.00910-23-s0004.pdf]

Table S2: Summary of data collection and atomic model statistics

**Data collection**			
Microscope	Krios G3 (ThermoFischer Scientific)		
Voltage (kV)	300		
Sample	HAd11K/rDSG2		HAd11K/rDGS2/rCD46
Acquisition software	SerialEM		EPU
Magnification<br>Unbinned pixel size	215,000x<br>0.325 Å/pixel		105,000x<br>0.42 Å/pixel
Camera	K2 Summit (Gatan Inc)		K3 (Gatan Inc)
Exposure time	6 s		2.3 s
Number of frames	40		60
Total dose ($e^{-}/Å^{2}$)	55		58
**Image processing**			
	HAd11K/one rDSG2	HAd11K/two rDSG2	HAd11K/one rDSG2/one rCD46
EMDB			
Symmetry	C1	C1	C1
Final number of Particles	105169	127522	198176
Map resolution in Å (FSC 0.143)	3.5	3.2	3.2
**Atomic model statistics**			
PDB			

Model resolution in Å (FSC 0.5)	3.8	3.3	3.6
Ramachandran favored (%)	97.3	96.3	96.9
Ramachandran outliers (%)	0.0	0.0	0
Rotamer outliers (%)	0.1	0	0.1
Rama Z score	-0.6	-0.8	-0.5
C-beta deviations	0	0	0
Rms on bond lengths	0.0041	0.0049	0.0031
Rms on bond angles	1.01	0.59	0.60
Clashscore	8.9	5.4	6.3
Molprobity score	1.60	1.54	1.53
